# Vulnerability to Nitrate Occurrence in the Spring Waters of the Sila Massif (Calabria, Southern Italy)

**DOI:** 10.3390/toxics10030137

**Published:** 2022-03-12

**Authors:** Ernesto Infusino, Ilaria Guagliardi, Simona Gaglioti, Tommaso Caloiero

**Affiliations:** 1 Department of Environmental Engineering (DIAm), University of Calabria, Via P. Bucci 41C, 87036 Rende, Italy; ernesto.infusino@unical.it (E.I.); simonagaglioti@gmail.com (S.G.); 2 National Research Council—Institute for Agricultural and Forest Systems in Mediterranean (CNR-ISAFOM), Via Cavour 4/6, 87036 Rende, Italy; tommaso.caloiero@isafom.cnr.it

**Keywords:** spring waters, hydrogeochemical characterization, statistical analysis, correlation, nitrate vulnerability

## Abstract

Knowledge of spring waters’ chemical composition is paramount for both their use and their conservation. Vast surveys at the basin scale are required to define the nature and the location of the springs and to identify the hydrochemical facies of their aquifers. The present study aims to evaluate the hydrochemical facies and the vulnerability to nitrates of 59 springs falling in the Sila Massif in Calabria (southern Italy) and to identify their vulnerability through the analysis of physicochemical parameters and the use of the Langelier–Ludwig diagram. A spatial analysis was performed by the spline method. The results identified a mean value of 4.39 mg NO_3_^−^/L and a maximum value of 24 mg NO_3_^−^/L for nitrate pollution in the study area. Statistical analysis results showed that the increase in electrical conductivity follows the increase in alkalinity values, a correlation especially evident in the bicarbonate Ca-Mg waters and linked to the possibility of higher nitrate concentrations in springs. These analyses also showed that nitrate vulnerability is dependent on the geological setting of springs. Indeed, the Sila igneous–metamorphic batholith, often strongly affected by weathering processes, contributes to not buffering the nitrate impacts on aquifers. Conversely, anthropogenic activities, particularly fertilization practices, are key factors in groundwater vulnerability.

## 1. Introduction

In spring waters, nitrogen is one of the principal biogenous elements. It naturally occurs in the environment or derives from anthropogenic input, commonly found in fertilizers and animal and human wastes. Usually, human activities are altering all biogeochemical cycles [1,2] and tend to particularly affect the natural nitrogen cycle either directly (e.g., industrial, residential, agricultural, farming discharge) or indirectly, by altering soil degradation processes [3,4,5,6,7,8]. Furthermore, the dynamics of the hydrogeological systems and their water resource quality can be affected by climate change [9,10,11,12].

Once nitrogen is in the environment and converted to nitrate form, it will completely dissolve in water and move easily with water to aquatic ecosystems, where it can cause undesirable effects. Indeed, epidemiological studies evidenced that high levels of nitrate in water are a human health concern [13], considering that nitrate is classified as a probable human carcinogen [14]. Several studies have been conducted on the potential onset of stomach or brain cancer in people caused by exposure to nitrates through drinking water [7,15,16,17]. More precisely, as a result of a reaction into an acid environment, when nitrates encounter the amines contained within food products, they produce nitrosamines, classified as carcinogenic substances [18]. Furthermore, nitrates have been demonstrated to be responsible for “blue baby syndrome” (methemoglobinemia) [19], especially in infants, whereby reduced intestinal acidity facilitates the proliferation of bacterial flora transforming nitrates into nitrites. The latter enter the blood and cause the modification of hemoglobin into methemoglobin, which is thus unable to transport oxygen to tissues, resulting in cyanosis. The “blue baby syndrome” occurs when methemoglobin levels exceed a 10% concentration in the blood [20].

According to the WHO, the maximum permissible concentration level of nitrates in drinking waters is 50 mg/L [21], while the standard level is 25 mg/L. The European Directive 98/83 CE sets the maximum concentration level for nitrates at 50 mg/L. At the national scale, the Legislative Decree No. 30/2009 [22] defines the criteria and procedure for assessing the chemical status of groundwater. It reports the environmental quality standards established at the community level for nitrates and identifies for a specific set of parameters, the threshold values adopted at the national level for the purpose of assessing the chemical status of groundwater. This decree sets the nitrate value for groundwaters at 50 mg/L for the determination of nitrate-vulnerable areas. Conversely, as to what concerns mineral waters [23], the limit value for nitrates is 45 mg/L, which becomes a precautionary 10 mg/L value for those mineral waters consumed during pregnancy and infancy.

For this health risk, studying spring water vulnerability to nitrates is paramount to solve possible aquifer contamination and/or have a clear historical picture for further evaluations. Deterministic or geostatistical methods are the right approach for studying the spatial structure of nitrate and for mapping spring water quality. Indeed, the application of these techniques to the interpretation of the relationship between the aquifer lithology and the chemical composition of spring water and of the contamination of groundwater has provided good results so far. Furthermore, other studies have carried out spring water analysis at a large scale, collecting considerable data and contributing to the water management decision making

Therefore, taking into account all previous considerations, from the intrinsic public health risk in water supply nitrate sources to geochemical and environmental implications of nitrate contents in water, the present work is aimed at classifying the spring water types of the Sila Massif (Calabria, southern Italy) according to their hydrogeochemical features, identifying the factors controlling mineral waters using spatial variables focused on lithological settings and determining the vulnerability to nitrate occurrence in the spring waters. The study area is characterized by many spring waters, and its groundwater is largely used for drinking purposes, thus representing a natural and socioeconomically important resource. However, few analyses on few samples have been carried out in the past years to assess the quality and the degradation processes affecting the aquifers of the Calabria region [24,25].

It is expected that the outcomes of this work could help to develop effective strategies for integrated water resource management, particularly by providing scientific evidence for decisions on and management of groundwater sources used for drinking water supply.

## 2. Study Area and Data

**Figure 1:**
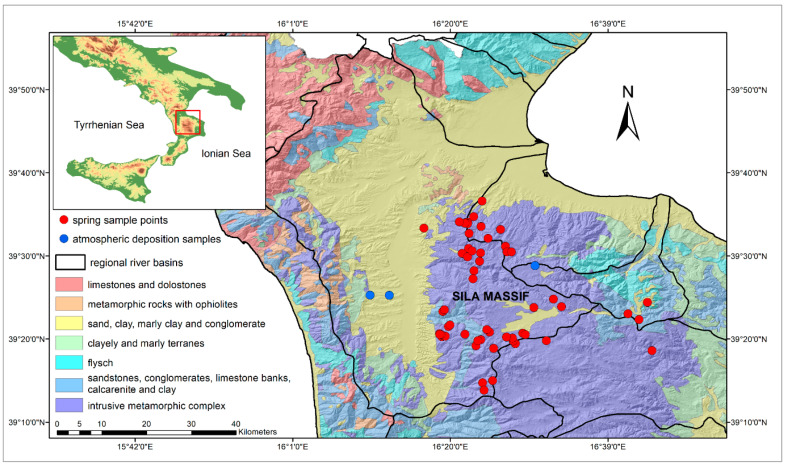
Map of the geographical and geological setting of the study area, with the locations of the samples.

The Sila Massif, located in the central-northern area of the Calabria region, is the study area (Figure 1). It extends for 150,000 hectares, which include mountains, plateaus, thick wooded areas, rivers and high-altitude lakes, through three different provinces of the region. From north to south it is usually divided into three subranges: Sila Greca, Sila Grande and Sila Piccola. The main rivers of Calabria originate from this massif, and some of these rivers feed artificial lakes [26].

The Sila Massif is part of a Paleogene orogenic belt (Calabrian Arc), thrusted over the Apennine Chain during Miocene times [27,28,29]. From a geological point of view, the area is a batholith formed by late Hercynian intrusive rocks and Paleozoic medium- to high-grade metamorphites, which are often strongly affected by weathering processes [30]: it underlies Mesozoic and Miocene to Quaternary sedimentary terranes [31,32]. The Paleozoic complex is composed mainly of paragneiss, biotite schists and grey phyllitic schists (with dominant quartz, chlorite and muscovite). The intense tectonics that molded the site, the subsequent and rapid uplifting affecting the area and an unfavorable set of climatic factors often reduced the crystalline rocks to sand [33,34].

The Quaternary is represented by Pliocene sediments, made up of light brown and red sands and gravels, blue-grey silty clays with silt interlayers, Pleistocene to Holocene alluvial sands and gravels and very small outcrops of Miocene carbonate rocks [35].

The dominant altitude of the central part of the Sila Massif is about 1300 m a.s.l., with the most representative peaks at almost 2000 m a.s.l.

Intense potato and cereal crops occupy the mountainous areas, while on the slopes, at the lower altitudes, there are pastures and forests of *Pinus laricio* (*Pinus nigra* subsp. *laricio calabrica*), many of which result from reforestation in the 1950s [36], and at the higher altitudes, beech (*Fagus sylvatica* L.) and silver fir (Abies alba) prevail.

Following the Köppen–Geiger classification [37], the climate of the study area can be identified as hot-summer Mediterranean, but due to its mountainous nature, colder winters with snow and cooler summer with some precipitation can be observed [38,39].

The study area is characterized by numerous springs with a density of 1.16/km^2^ but of reduced flow rate (many of which have a flow rate lower than 1 L/s), typical of schistose–granitic settings which are almost impermeable.

In this survey, 59 spring waters were identified and sampled in the Sila Massif (Figure 1) together with 3 atmospheric deposition locations. For each site, an identification form was compiled accompanied by photographic material and contained all the initial information collected. The sampling was carried out between the months of February and May 2016, a period in which the springs’ maximum flow rates are generally recorded due to aquifer recharge following snow melting at high altitude and/or more simply following rainfall.

## 3. Methodology

### 3.1. Laboratory Analysis

The samples collected were analyzed at the Genesis of Pollutants in the Water Cycle (GICA) laboratory at the University of Calabria. To evaluate the geochemical and hydrodynamic characteristics of the springs and in order to classify the investigated areas [40,41,42], a consolidated methodological procedure in geochemical approach was followed, analyzing the so-called labile parameters (temperature, pH and specific EC compensated at 20 °C) at the time of sampling by using portable apparatus including a pH meter and a conductivity meter and analyzing the main cations and anions once the samples reached the laboratory in clean high-density polyethylene (PE-HD) bottles with screw caps.

The major cations (Ca^2+^, Mg^2+^, Na^+^, K^+^) were determined by using the atomic absorption spectroscopy (AAS) method, major anions (NO_3−_, SO_4_^2−^, Cl^−^, HCO_3_^−^) were determined by using the UV spectrophotometry method, alkalinity with determined by titration with 0.1 N HCl on unfiltered samples (expressed as mg HCO_3_^−^/L) and hardness was determined by using complexometric EDTA titration.

Total dissolved solutes (TDS) expressed in mg/L is calculated as the sum of all major anions (Cl^−^, NO_3_^−^, SO_4_^2−^ and alkalinity as HCO_3_^−^) and cations (Na^+^, K^+^, Mg^2+^ and Ca^2+^).

A quality control analysis was performed evaluating sampling and analytical duplicates. Analytical precision was calculated by the 20% analysis (in duplicate) of randomly chosen samples and was found to be within accepted international standards (<5%).

### 3.2. Facies Classification Analysis

In literature, there are several methods to identify the dominant facies via a comparison of the most salient chemical characteristics, including by means of graphic analysis such as qualitative diagrams, which offer a good visual interpretation but no indication of the actual water mineralization, and quantitative diagrams, which, instead, provide an indication of mineralization [43].

The analyses carried out have allowed classifying the spring waters according to Italian Legislative Decree No. 31/2001, which categorizes waters as follows:

(a) TDS (mg/L):
  - 0 < TDS < 50—very low mineral content water (or light mineral water);
  - 50 < TDS < 500—low mineral content water;
  - 500 < TDS < 1500—medium mineral content water;
  - TDS > 1500—waters rich in mineral salts.
(b) Hardness (H) due to the presence of calcium and magnesium ions expressed in French degrees (1° f = 10 mg/L = 10 mg/kg) or the equivalents of CaCO_3_:
  - H < 15—light or soft water;
  - 15 < H<30—average hard water;
  - H > 30—hard water.

To determine the tendency of water to attack and solubilize certain minerals contained in rocks and soils, the aggressiveness index I_A_ proposed by the American Water Works Association [44,45] was determined. This index, a simplified form of the Langelier Index, valid within the temperature range 4.5–26.5 °C, is expressed by the formula:

(1) $$I_{A}=pH+{log}_{10}\left(A\cdot H\right)$$

where A is the total alkalinity (mg/L HCO_3_) and H is the calcium hardness (mg/L CaCO_3_).

According to this index, the more aggressive the water is, the lower the value I_A_:

- I_A_ < 10—aggressive water;
- 10 < I_A_<12—moderately aggressive water;
- I_A_ > 12—nonaggressive water.

In order to graphically visualize the water chemistry, various diagrams have been developed over the years [46,47]. Among others, in this paper, the hydrochemical facies were determined according to the Langelier–Ludwig (LL) square diagram [48], which has been largely applied in literature [49,50,51,52]. In particular, Langelier and Ludwig proposed a diagram in which rectangular coordinates are used to represent patterns and correlations between major cations and anions for multiple samples, thus allowing separation between Ca from Mg facies and Cl from SO_4_ facies. More precisely, with the LL diagram, the water classification is ensured by positioning the water sample in one of the four sectors of which the diagram is composed:

- Chloride–sulfate Ca-Mg waters;
- Chloride–sulfate alkaline waters;
- Bicarbonate alkaline waters;
- Bicarbonate Ca-Mg waters.

The identified ion concentrations are recalculated from mg/L to meq/L. Therefore, knowing all meq/L ion concentrations, the dimensionless value *R* can be calculated for each *i*-th cation *C* or anion *A*, according to the following relationships, and plotted on the LL diagram:

(2) $$RC_{i}=\frac{50\cdot C_{i}}{\sum C_{tot}}$$

(3) $$RA_{i}=\frac{50\cdot A_{i}}{\sum A_{tot}}$$

The alignment of the sample points into the different sectors allows the identification of possible phenomena such as mixing or evolutionary processes of water [25].

### 3.3. Atmospheric Depositions Input Analysis

The presence of nitrogen compounds in the air is due both to natural causes (soil erosion, volcanic eruptions, etc.) and anthropogenic activities (fires, industrial plants, motor vehicles, heating, nitrogenous fertilizers). Nitric acid (HNO_3_) in the atmosphere mainly results from the transformation of NO_2_ reacting with free radicals. NO_2_ in turn is formed by the reaction of NO with ozone. Nitric acid has a high deposition rate. In an aqueous medium, this compound turns into nitrite and nitrate ions. Nitric acid reacts with ammonia to form ammonium nitrate. Ammonium nitrate is in an equilibrium state with nitric acid and ammonia for relative humidity lower than 60%. Ammonium nitrate, which has low sedimentation, can be transported away from its originating source, and if humidity exceeds 60%, it turns back into nitrate and ammonia, contributing to the formation of acidity even in distant places.

To quantify the contribution of background nitrogen concentration from the atmosphere, total depositions at three different sites (Figure 1) were measured. Specifically, the three sites are denominated according to their location: San Antonello in Montalto Uffugo (175 m a.s.l.), Settimo in Montalto Uffugo (250 m a.s.l.) and Bonis (1090 m a.s.l.).

### 3.4. Data Analysis

A preliminary analysis of the dataset was performed through the box plot test in order to identify probable outliers.

Spatial interpolation is the procedure to predict the value of attributes at unobserved points within a study region using existing observations [53].

Spatial interpolation covers a variety of methods including trend surface models, Thiessen polygons, kernel density estimation, inverse distance weighting, splines and kriging. Among these several methods, mostly used in environmental analysis, in water resources management and in hydro-geochemical forecasting, the spline interpolation method was adopted for the analysis of the spatial variation of nitrate concentration in the spring waters of the Sila Massif. The spline interpolation model was chosen since the distribution of the sampled points did not allow a homogeneous coverage of the study area, and therefore the model that was least affected by the effect of inhomogeneity in the distribution and spacing between the points was applied. In fact, the spline method can generate sufficiently accurate surfaces from only a few sampled points, and the generated surfaces retain small features.

Spline interpolation is a deterministic interpolation method that fits a mathematical function through input data to create a smooth surface [54,55]. Splines are piecewise polynomial functions that are fitted together to provide a complete, yet complex, representation of the surface between the measurement points. Functions are fitted exactly to a small number of points while, at the same time, ensuring that the joins between different parts of the curve are continuous and have no disjunctions.

Spline interpolation creates a surface that passes through the control points and has the least possible changes in slope at all the points. This method uses a mathematical function with input points:

(4) $$Q\left(x,y\right)=\sum_{i=1}^{N}A_{i}d_{i}^{2}logd_{i}+a+bx+cy$$

(5) $$d_{i}^{2}={\left(x-x_{i}\right)}^{2}+{\left(y-y_{i}\right)}^{2}$$

where *x* and *y* are the coordinates of the point to be interpolated, and *x_i_* and *y_i_* are the coordinates of the control point.

This function minimizes the overall surface curvature, resulting in a smooth curvature passing exactly through the input points.

A regularized spline yields a smooth surface and smooth first derivatives. With the regularized option, higher values used for the weight parameter produce smoother surfaces. The values entered for this parameter must be equal to or less than zero. Typical values that may be used are 0, 0.001, 0.01, 0.1 and 0.5. A tension spline is flatter than a regularized spline of the same sample points, forcing the estimate to stay closer to the sample data. It produces a surface more rigid according to the character of the modeled phenomenon. With the tension option, higher values entered for the weight parameter result in somewhat coarser surfaces, but surfaces that closely conform to the control points. The values entered must be equal to or greater than zero. The typical values are 0, 1, 5 and 10. The tension spline was evaluated as the best method for generating surfaces that vary gently, such as modeling of aquifer levels or groundwater pollution concentrations.

To determine the interpolation errors, the values of RMSE, %RMSE and MRE were assessed by Equation (6), Equation (7) and Equation (8), respectively. Values of RMSE and MRE close to zero indicate a good estimate of the model used to assess the unknown parameters:

(6) $$RMSE=\sqrt{\frac{\sum_{j=1}^{n}{\left(x{\left(P\right)}_{j}-x{\left(m\right)}_{j}\right)}^{2}}{n}}$$

(7) $$\%RMSE=\frac{RMSE}{\bar{X}}*100$$

(8) $$MRE=\frac{1}{n}\sum_{i=1}^{n}\frac{\left|z^{*}\left(x_{i}\right)-z\left(x_{i}\right)\right|}{z\left(x_{i}\right)}$$

where *x*(*P*) is the estimated value of each component, *x*(*m*) is the measurement of water parameter, *n* is sample number, *X* is the mean of a measured parameter, *z*(*x_i_*) is the observed value at location *i*, z*(*x_i_*) is the interpolated value at location *i* and *n* is the sample size.

The spatial analysis tool used for this analysis was ArcGIS 9.3.1 software.

## 4. Results and Discussion

A summary of physicochemical parameters of spring water samples and their statistics is provided in Table 1.

The range of pH of spring waters is between 5.46 and 8.6 (mean = 6.86).

The mean water temperature of the analyzed springs is 10.51 °C with variability between 6–16 °C and a decreasing trend with altitude *Z* (m a.s.l.) according to the following correlation:

(9) T(°C)=−0.0052Z+16.257 

The thermal gradient of −0.0052 °C/m indicates the presence of relatively shallow aquifers, influenced by the external air temperature. The spring temperature seems to be mainly influenced by vertical zonation because there are no statistically significant differences in temperature between springs located in different geological formations at the same altitude. In addition, the behavior of temperature vs. altitude is probably due to recharge from snow melting during springtime, which can promote water temperature decrease, especially in springs located at higher altitudes.

Electrical conductivity ranged between 51.3 and 710.61 μS/cm with a mean of 179.37 μS/cm.

The coefficient of variation (CV, %) representing the degree of scattering, showed a wide range among ions, with values for Na^+^ (73.7%), Cl^−^ (66.26%), SO_4_^2−^ (216.62%), Ca^2+^ (88.48%), Mg^+^ (69.7%), K^+^ (37.3%), HCO_3_^−^ (90.34%) and NO_3_^−^ (124.11%) (Table 1).

The major cation concentrations were detected for Ca^2+^ and Na^+^, while the major anions ones were for HCO_3_^−^ and Cl^−^. The nitrate (NO_3_^−^) concentration had a mean value of 4.38 mg/L, and no sample exceeded the threshold value of 50 mg/L of WHO [21] and Italian Legislative Decree [22] recommendations. These results are in accordance with previous surveys, where the nitrate concentration did not generally exceed 2 mg/L in uncontaminated spring water aquifers [56,57]; anyway, in case of contamination, nitrates can reach extremely high levels. Nitrate pollution is increasing in many countries [58]. One example is in the Campania region (Italy), where nitrate values exceeding 200 mg/L in variable concentrations both in space and time have been found in spring waters and wells [59].

**Figure 2:**
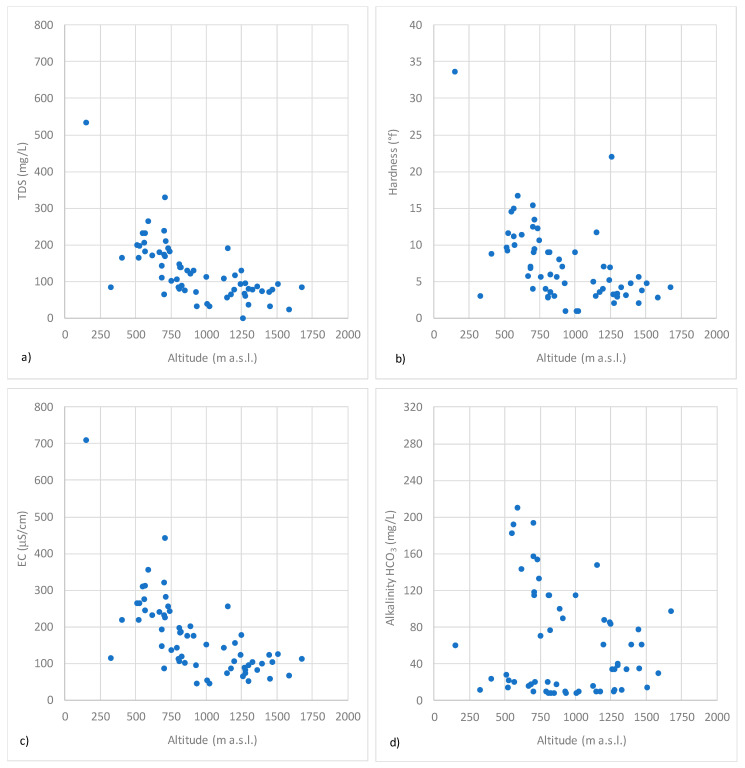
Bivariate plots of TDS (**a**), H (**b**), EC (**c**) and A (**d**) vs. altitude.

The spring water samples can be classified as very low mineral content (TDS < 50 mg/L) and low mineral content (50 < TDS < 500 mg/L), with a single sample that reaches the TDS value of 533 mg/L. These results indicate that the geochemistry of spring waters in the Sila Massif is strongly affected by the mineralogical composition of the local rocks and controlled by hydrolysis of sodium and/or calcium silicate minerals. The samples generally present an increase in low TDS with a decrease in altitude (Figure 2a). As regards the hardness, a trend similar to that of TDS can be observed (Figure 2b). With few exceptions, the springs analyzed can be classified as light or soft waters, with a hardness value lower than 15 °f. There is a good correlation between hardness and TDS.

**Figure 3:**
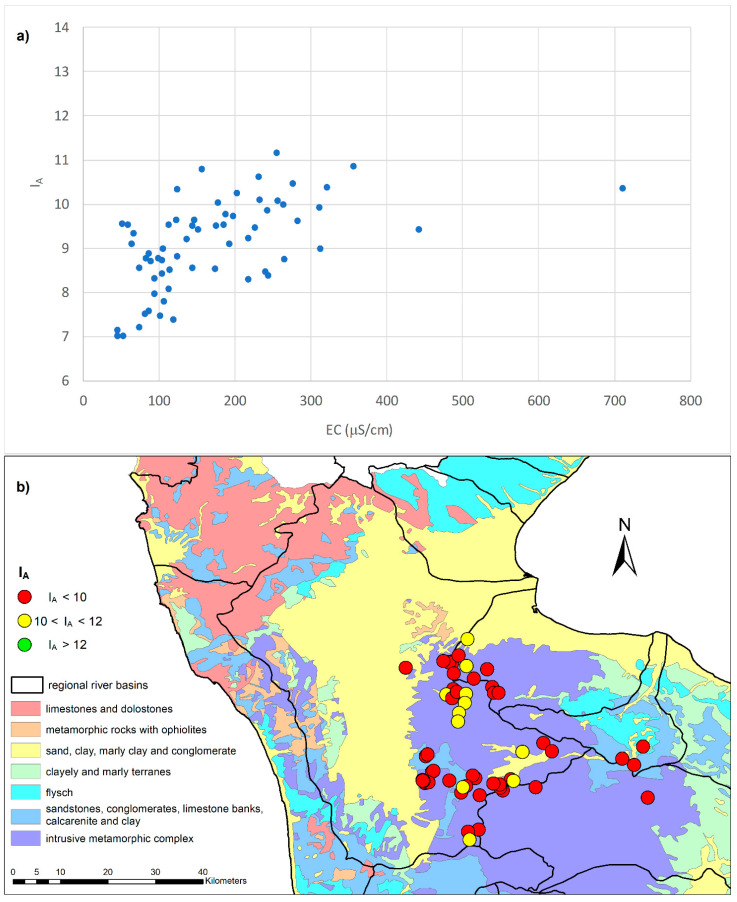
Bivariate plots of I_A_ vs. EC (**a**) Bivariate plots of I_A_ vs. EC and (**b**) I_A_ classification of the spring waters according to geology.

According to the results of the aggressiveness index I_A_, it was possible to classify 78% of the analyzed springs as aggressive (I_A_ < 10) and the remaining 22% as moderately aggressive (10 < I_A_ < 12), the latter having a higher conductivity than the former. Figure 3 shows the I_A_ classification of the spring waters.

Aggressiveness is mainly due to the presence of carbon dioxide (CO_2_), in the form of carbonic acid, as well as the presence of sulfates and nitrates which reduce the water pH. The acids, not being buffered by the acid rocks of the aquifers, act as a solubilizing agent on some minerals, which are washed out.

A correlation matrix of hydrochemical parameters was constructed and is shown in Table 2.

Because geological properties are closely related to characteristics of natural springs, including discharge, chemical composition, and temperature, hydrochemical parameter analysis between the occurrence of spring water and geological factors can be useful. Indeed, extensive monitoring campaigns are necessary, which can determine the natural cycle of spring waters resulting from the interaction between rocks, atmosphere, mixing with older aquifers and the effects of human activities. Groundwaters, because of their interaction with various matrices, become rich in gases such as carbon dioxide, oxygen and nitrogen; carbonic acid salts and strong bases, e.g., calcium, magnesium, sodium and potassium carbonates; and strong acid salts and strong bases, such as sulfates, chlorides, calcium, magnesium, sodium and potassium nitrates.

Accordingly, in this survey, major cations including Na^+^ and Mg^2+^ showed a linear relationship with alkalinity. This can be explained by the acidic hydrolysis of minerals. The hydrolysis reaction consumes water and acid that might have originated from CO_2_ and increases the pH, alkalinity and cation concentration of water.

Further evaluations can be detected by observation of electrical conductivity values according to altitude for each zone (Figure 2c): although the altitude decreasing is combined with the conductivity increasing (with the exception of the three spring waters in which higher NO_3__−_ concentration was detected), this last one remains low, thus evidencing a typical behavior of the igneous lithology and the presence of relatively shallow aquifers affected by atmospheric depositions [60].

**Figure 4:**
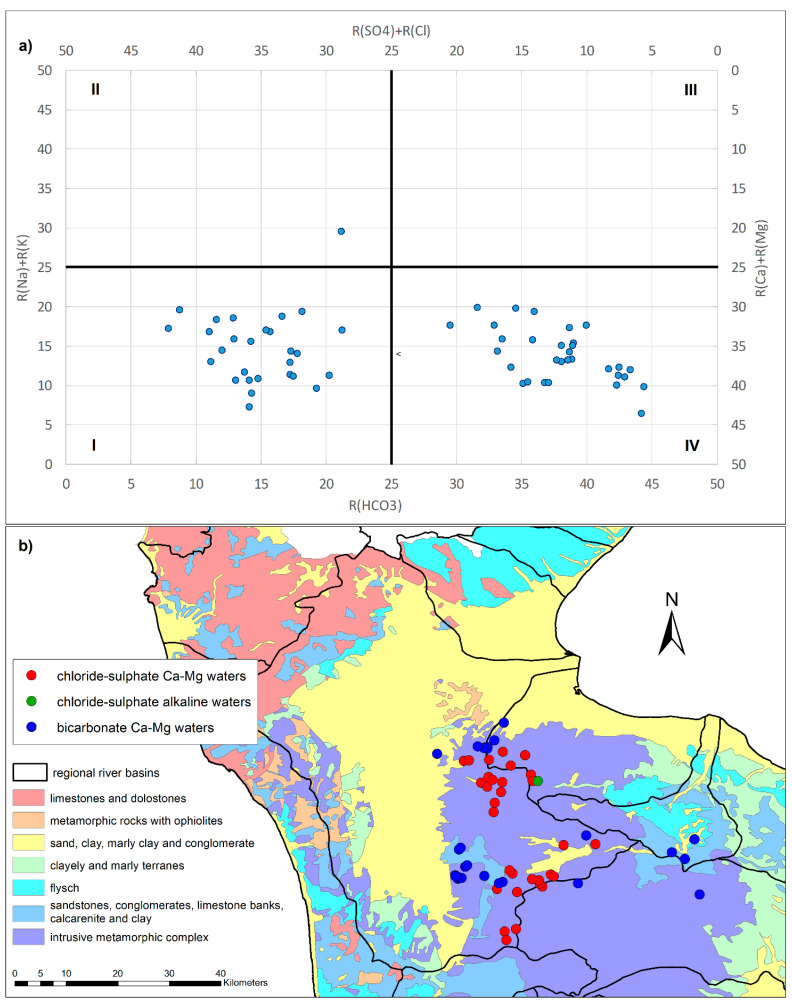
Langelier–Ludwig (LL) diagram (**a**) and classification of the spring waters (LL quadrants) according to geology (**b**).

The Langelier–Ludwig (LL) square plot, obtained by positioning the representative points of the individual springs within the four standard quadrants, is represented in Figure 4a.

**Figure 5:**
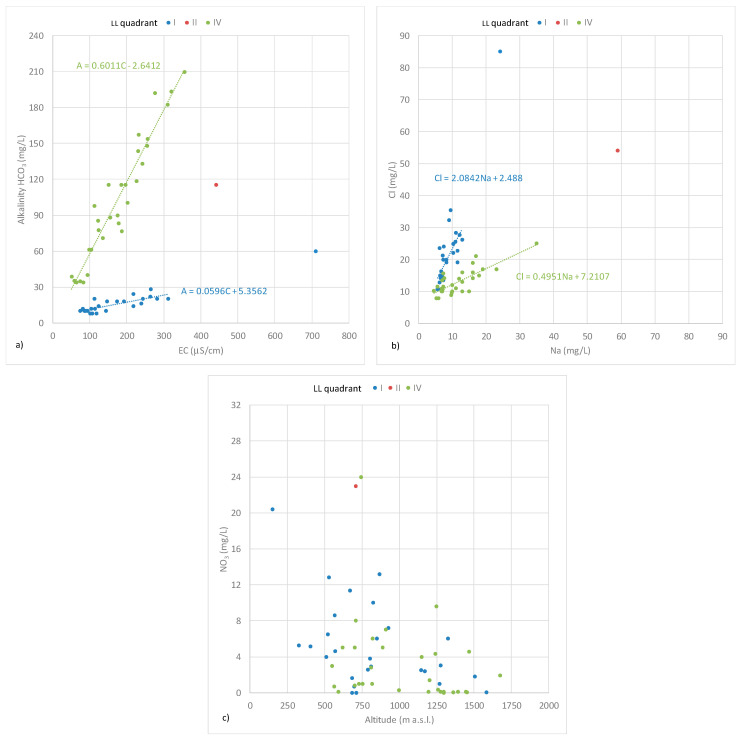
(**a**) Correlations between EC and A, (**b**) Na^+^ and Cl^−^ and (**c**) NO_3_^−^ and altitude according to classification of water springs in LL diagram.

According to their chemical compositions, the samples fall into three of the four quadrants of the LL diagram:

- Chloride–sulfate Ca-Mg waters (I quadrant). The waters belonging to these facies mainly spring from the slopes forming the crown of the Sila Massif (Figure 4b). These springs fall into geological units made up of acid rocks (acid granulites, biotic gneisses). They have a low alkalinity which is almost independent of altitude. For them, good correlations between conductivity EC (µS/cm) and alkalinity A in terms of HCO_3_ (mg/L) and between chloride and sodium ions were observed.
- Chloride–sulfate alkaline waters (II quadrant). A limited number of springs belong to these facies, falling in metamorphic geological units located at the extreme offshoots of the investigated area, arising from metamorphic rocks. An exception is a spring in another setting (the municipality of Acri) classifiable within these facies due to its high content of sodium, chlorides, etc., although the aquifer rocks are granite like those of the IV quadrant. The waters are rich in sodium and potassium ions and show a low calcium and magnesium content. Alkalinity does not differ from that described for the I quadrant.
- Bicarbonate Ca-Mg waters (IV quadrant). They are the springs arising in the central part of the Sila Massif, from acid rocks (acid granulites, biotic gneisses, granites, granodiorites, magmatites). Alkalinity A increases with decrease in altitude Z. For these waters, an excellent correlation between conductivity EC (µS/cm) and alkalinity A was found (Figure 5a).

A good correlation between chloride and sodium ions was observed (Figure 5b).

Waters with alkalinity close to 40 mg/L show a change in their gradient, becoming very similar to that of the I quadrant.

The sampled waters located in the north and east of the Sila Massif, which fall in the facies of chloride–sulfate alkaline waters, show a NO_3_^−^ concentration above 10 mg/L. This value is considerably higher than the other samples of about 2 mg/L found in springs unaffected by anthropogenic activities.

The increase in EC follows the increase in alkalinity values, a correlation especially evident in the bicarbonate Ca-Mg waters and linked to the possibility of higher nitrate concentrations in springs (Figure 5a).

Generally, the analyzed waters have a nitrate content lower than 5 mg/L, especially the bicarbonate Ca-Mg waters (IV quadrant), which show a mean NO_3_^−^ content of 2.45 mg/L, as well as the chloride–sulfate alkaline waters (II quadrant) with a mean NO_3_^−^ content of 1.8 mg/L. Instead, the chloride–sulfate Ca-Mg waters (I quadrant) are generally richer with a mean NO_3_^−^ content of 4.8 mg/L with a maximum value of 13 mg/L.

The results of statistical data analysis have detected the absence of outliers. As shown in Figure 5c, three water springs arising at hilly altitudes, falling in the municipalities of Acri and San Demetrio Corone, exhibit an anomalous behavior compared to the other analyzed water springs in terms of conductivity and presence of ions (Cl^−^, Na^+^, etc.). These three anomalous spring waters have nitrate concentrations ranging between 20 and 24 mg/L. These samples also present an anomalous content of the other analyzed parameters, such as a high conductivity, indicating probable aquifer contamination mainly due to widespread wild farming and pastures in the area, most practiced in the municipalities in which they fall. This factor could induce a greater exposure to anthropogenic contaminants and result in higher nitrate concentration.

Table 3 shows the mean concentrations of NH_4_^+^, NO_2_^−^ and NO_3_^−^ recorded in the three sampling sites for the atmospheric deposition analysis. They allow us to define the extent of the atmospheric contribution into soil, considering that the concentration trend is seasonal with the maximum in spring. From previous studies, the nitrate concentration in the Bonis area (Sila Massif) is about 2 mg/L [61]; it shows lower concentrations than those found in the Crati valley (Sant’Antonello and Settimo) in Montalto Uffugo.

Factors that may account for the higher deposition rates in these sites include higher rainfall amounts, local atmospheric inputs of N from volatilization of ammonia from fertilizers and animal wastes and N from the combustion of fossil fuels.

Cross-validation results for predicted versus measured nitrate values evidenced a good performance of the spline model in the study area. In fact, both MRE and RMSE values showed that the spline model can predict the data accurately.

**Figure 6:**
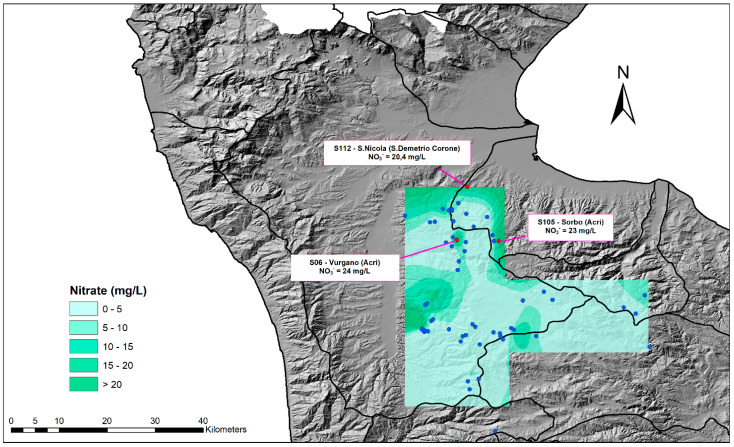
Spatial distribution of nitrates in the study area.

Figure 6 shows the spatial distribution of nitrates in the study area mapped through the spline method in five different ranges of values. The results were represented using the digital terrain model in the background.

The obtained results were consistent with other studies that emphasized the spline method was one of the most suitable techniques for mapping nitrate concentrations in spring water. Indeed, the use of the spline method for water quality spatial analysis has been verified by numerous scientists. Guo et al. [62] investigated the five typical spatial interpolation methods, namely kriging, natural neighborhood, IDW, spline and trend surface, and found that the IDW and spline methods are appropriate for platform and small undulating areas. The national neighbor method and spline method provided the most accurate estimates of nitrates in the aquifer in the Qazvin plain [63]. The results of Zabihi et al. [64] indicate that the spline method is a good estimator of groundwater spring potential in the Iran area. The performance of the spline method was excellent in a case study in the Khalkhal region (Iran) [65].

The spatial distribution of nitrates provides some insight into the geochemical processes during the evolution of groundwater in the study area. The maximum level of nitrate concentrations was located in springs at lower altitudes in the north and western parts of the study area, in which lower precipitation and more intense agricultural activities occur compared to the eastern area. This factor could induce a greater exposure to anthropogenic contaminants and result in higher nitrate concentration.

The minimum level of nitrate, instead, can be attributed to the high-altitude area, which has no human activity and in which the lithological distribution may give an explanation for these values, as mentioned above.

In addition, in the Sila Massif, it is necessary to underline the subsequent reforestations since the postwar period and the related cleaning and maintenance activities of the woods that have deprived the subsoil of the organic substrate necessary for denitrifying bacterial colonies.

The study area was selected for its characteristics, along with the type of land use and human pressure, which are common to other wide Mediterranean areas, and therefore, despite its relatively small extension, it can well represent wider territories.

Moreover, the investigated area represents an extraordinary case of geological homogeneity, with the occurrence of purely acid metamorphic rocks. In it, there are few and no significant anthropogenic presences, and the only inputs that contribute to the nitrate vulnerability in spring waters are attributable to the agricultural uses of soil and especially to forestry as well as to atmospheric depositions.

In addition, in the study area, investigations for vulnerability to nitrate occurrence in spring waters are scarce. The present survey confirmed the importance of assessing nitrate pollution as an environmental issue with multiple implications in terms of water quality deterioration, biodiversity loss, human health problems, global carbon-cycle alterations and climate change.

## 5. Conclusions

Springs perform numerous tasks, including ensuring sources of potable water and providing recreational, ecological and cultural value; moreover, they provide a way to assess groundwater quality since they are the connection between groundwater and surface water and give direct information about the state of groundwater in the aquifers that feed them.

The main focuses of this survey were to classify the spring water types of the Sila Massif (Calabria, southern Italy) according to their hydrogeochemical features and thus identify the factors controlling mineral waters using spatial variables focused on lithological settings and finally determine the vulnerability to nitrate occurrence in the spring waters.

The characterization of the waters of 59 springs in the study area has shown that they are strongly affected by the geological nature of the rocks of the aquifers (granites, magmatites, acid granulites, biotic gneiss, etc.). Indeed, the waters are generally poor in mineral salts (very low mineral content waters) with low calcium and magnesium content (soft).

The hydrochemical facies of the aquifers from which the water springs draw, according to the classification proposed by Langelier–Ludwig, can be classified as chloride–sulfate alkaline waters, those arising mostly from the mountainous slopes that crown the Sila Massif, and bicarbonate Ca-Mg waters that flow mostly in the central part of the Sila.

According to these considerations, most of the sampled points, having lower nitrate values, demonstrate the overall good quality of the spring waters in the Sila Massif. Therefore, the monitoring of springs in the Sila Massif revealed the scarce vulnerability to a potential alteration of their groundwaters by nitrates.

## Figures and Tables

**Table 1:** Basic statistics for analyzed water parameters.

Parameter	Min	Max	Mean	Median	Lower Quartile	Upper Quartile	Standard Deviation	Skewness	Kurtosis	CV (%)
pH	5.46	8.6	6.86	6.82	6.5	7.24	0.58	0.36	0.51	8.48
EC (µS/cm)	51.3	710.61	179.37	152	102.9	236	110.7	2.2	8.13	61.72
H (°f)	2.01	33.6	7.7	6	3.7	9.8	5.5	2.23	7.7	71.35
TDS (mg/L)	0.12	533	132.73	113	78.11	175.9	83.97	2.1	7.92	63.27
Ca^2+^ (mg/L)	3.61	110	16.42	14.4	8.61	20	14.53	4.77	29.92	88.48
Mg^2+^ (mg/L)	0.97	20.47	7.92	6.8	3.1	12.07	5.5	0.6	−0.81	69.7
K^+^ (mg/L)	0.009	2.92	1.46	1.56	1.1	1.85	0.55	−0.3	0.25	37.3
Na^+^ (mg/L)	4.51	59	11.27	9.05	6.96	12.5	8.3	3.87	19.25	73.7
HCO_3_^−^ (mg/L)	8	209.82	63.08	35.12	17	98.94	56.99	0.97	−0.11	90.34
Cl^−^ (mg/L)	7.80	85.12	18.07	14.9	10.72	21.14	11.97	3.62	17.41	66.26
SO_4_^2−^ (mg/L)	0.07	25	2.08	0.6	0.31	0.9	4.52	3.67	14.66	216.62
NO_3_^−^ (mg/L)	0.06	24	4.38	2.8	0.7	5.65	5.38	2.14	5	124.11

CV (%) = coefficient of variation; EC = electrical conductivity; H = hardness; TDS = total dissolved solutes.

**Table 2:** Pearson correlation coefficients for analyzed parameters.

	EC	TDS	pH	H	Ca_2_^+^	Mg_2_^+^	Na^+^	K^+^	Cl^−^	SO_4_^2−^	NO_3_^−^	A	Altitude
EC	**1**												
TDS	**0.99**	**1**											
pH	0.11	0.08	**1**										
H	**0.82**	**0.78**	0.12	**1**									
Ca_2_^+^	**0.85**	**0.85**	0.17	**0.83**	**1**								
Mg_2_^+^	**0.78**	**0.77**	0.14	**0.71**	**0.53**	**1**							
Na^+^	**0.71**	**0.70**	0.01	0.47	0.45	**0.52**	**1**						
K^+^	**0.51**	**0.51**	0.05	0.37	0.39	0.36	0.23	**1**					
Cl^−^	**0.76**	**0.76**	−0.04	**0.53**	**0.68**	0.33	**0.56**	**0.63**	**1**				
SO_4_^2−^	0.32	0.32	−0.08	0.18	0.19	0.18	**0.60**	−0.03	0.22	**1**			
NO_3_^−^	**0.57**	**0.57**	−0.10	0.31	0.38	0.29	**0.52**	0.33	**0.65**	0.26	**1**		
A	0.44	0.43	0.15	0.48	0.41	**0.59**	**0.52**	−0.11	−0.07	0.37	0.02	**1**	
Altitude	**−0.67**	**−0.68**	0.05	**−** **0.53**	−0.48	**−** **0.66**	−0.43	**−** **0.56**	**−** **0.50**	−0.15	−0.43	−0.16	**1**

Coefficients are significant at the 0.05 level (2-tailed), and those higher than 0.5 are shown in bold.

**Table 3:** Mean concentrations of NH_4_^+^, NO_2_^−^ and NO_3_^−^ detected in the three sampling sites.

Measurement Site	Coordinates	NH_4_^+^ (mg/L)	NO_2_^−^ (mg/L)	NO_3_^−^ (mg/L)
Sant’Antonello in Montalto Uffugo	39°28′47″ N	0.8296	0.0462	3.3287
	16°14′28″ E			
Settimo in Montalto Uffugo	39°25′15″ N	0.6304	0.0150	1.1121
	16°12′38″ E			
Bonis	39°28′50″ N	2.1325	0.0249	1.8664
	16°30′12″ E			

## Data Availability

The data presented in this study are available on request.

## References

[1] Kalvāns A., Popovs K., Priede A., Koit O., Retiķe I., Bikše J., Dēliņa A., Babre A. (2021). Nitrate vulnerability of karst aquifers and associated groundwater-dependent ecosystems in the Baltic region. Environ. Earth. Sci..

[2] Zuzolo D., Cicchella D., Lima A., Guagliardi I., Cerino P., Pizzolante A., Thiombane M., de Vivo B., Albanese S. (2020). Potentially toxic elements in soils of Campania region (Southern Italy): Combining raw and compositional data. J. Geochem. Explor..

[3] Buttafuoco G., Guagliardi I., Tarvainen T., Jarva J. (2017). A multivariate approach to study the geochemistry of urban topsoil in the city of Tampere, Finland. J. Geochem. Explor..

[4] Cicchella D., Zuzolo D., Albanese S., Fedele L., di Tota I., Guagliardi I., Thiombane M., de Vivo B., Lima A. (2020). Urban soil contamination in Salerno (Italy): Concentrations and patterns of major, minor, trace and ultra-trace elements in soils. J. Geochem. Explor..

[5] Fernández-Martínez M., Corbera J., Domene X., Sayol F., Sabater F., Preece C. (2020). Nitrate pollution reduces bryophyte diversity in Mediterranean springs. Sci. Total Environ..

[6] Galloway J.N., Townsend A.R., Erisman J.W., Bekunda M., Cai Z., Freney J.R., Martinelli L.A., Seitzinger S.P., Sutton M.A. (2008). Transformation of the nitrogen cycle: Recent trends, questions, and potential solutions. Science.

[7] Kazakis N., Matiatos I., Ntona M.M., Bannenberg M., Kalaitzidou K., Kaprara E., Mitrakas M., Ioannidou A., Vargemezis G., Voudouris K. (2020). Origin, implications and management strategies for nitrate pollution in surface and ground waters of Anthemountas basin based on a δ^15^N-NO_3_− and δ^18^O-NO_3_−isotope approach. Sci. Total Environ..

[8] Xiao Q., Wu K., Shen L. (2016). Nitrate fate and origin in epikarst springs in Jinfo Mountain area, Southwest China. Arab. J. Geosci..

[9] Buttafuoco G., Caloiero T., Guagliardi I., Ricca N. Drought assessment using the reconnaissance drought index (RDI) in a southern Italy region. Proceedings of the 6th IMEKO TC19 Symposium on Environmental Instrumentation and Measurements.

[10] Caloiero T., Guagliardi I. (2021). Climate change assessment: Seasonal and annual temperature analysis trends in the Sardinia region (Italy). Arab. J. Geosci..

[11] Mas-Pla J., Menció A. (2019). Groundwater Nitrate Pollution and Climate Change: Learnings from a Water Balance-Based Analysis of Several Aquifers in a Western Mediterranean Region (Catalonia). Environ. Sci. Pollut. Res..

[12] Pellicone G., Caloiero T., Guagliardi I. (2019). The De Martonne aridity index in Calabria (Southern Italy). J. Maps.

[13] European Environment Agency (EEA) (2003). Europe’s Environment: State of the Environment Report No. 3/2003.

[14] Darvishmotevalli M., Moradnia M., Noorisepehr M., Fatehizadeh A., Fadaei S., Mohammadi H., Salari M., Jamali H.A., Daniali S.S. (2019). Evaluation of carcinogenic risks related to nitrate exposure in drinking water in Iran. MethodsX.

[15] Mohammadi A.A., Zarei A., Majidi S., Ghaderpoury A., Hashempour Y., Saghi M.H., Alinejad A., Yousefi M., Hosseingholizadeh N., Ghaderpoori M. (2019). Carcinogenic and non-carcinogenic health risk assessment of heavy metals in drinking water of Khorramabad, Iran. MethodsX.

[16] Rezaei H., Jafari A., Kamarehie B., Fakhri Y., Ghaderpoury A., Karami M.A., Ghaderpoori M., Shams M., Bidarpoor F., Salimi M. (2018). Health-risk assessment related to the fluoride, nitrate, and nitrite in the drinking water in the Sanandaj, Kurdistan County, Iran. Hum. Ecol. Risk Assess. Int. J..

[17] Rehman J.U., Ahmad N., Ullah N., Alam I., Ullah H. (2020). Health Risks in Different Age Group of Nitrate in Spring Water Used for Drinking in Harnai, Balochistan, Pakistan. Ecol. Food. Nutr..

[18] International Agency for Research on Cancer (2010). IARC Monographs on the Evaluation of Carcinogenic Risks to Humans. Volume 94—Ingested Nitrate and Nitrite, and Cyanobacterial Peptide Toxins.

[19] Massoudinejad M., Ghaderpoori M., Jafari A., Nasehifar J., Malekzadeh A., Ghaderpoury A. (2018). Data on nitrate and nitrate of Taham dam in Zanjan (Iran). Data Brief..

[20] Knobeloch L., Salna B., Hogan A., Postle J., Anderson H. (2000). Blue babies and nitrate-contaminated well water. Environ. Health Perspect..

[21] World Health Organization (WHO) (1993). Guidelines for Drinking-Water Quality.

[22] Decreto Legislativo (D.Lgs.) 16 Marzo 2009, n. 30 (Pubblicato nella Gazz. Uff. 4 Aprile 2009, n. 79). Attuazione della direttiva 2006/118/CE, Relativa Alla Protezione Delle Acque Sotterranee Dall’inquinamento e dal Deterioramento. https://www.gazzettaufficiale.it/atto/serie_generale/caricaDettaglioAtto/originario?atto.dataPubblicazioneGazzetta=2013-04-04&atto.codiceRedazionale=13G00075.

[23] Decreto Legislativo (D.Lgs.) 2 Febbraio 2001, n. 31. (Pubblicato nella Gazz. Uff. 3 Marzo 2001, n. 52). Attuazione della Direttiva 98/83/CE, Relativa alla Qualità delle Acque Destinate al Consumo Umano. https://www.camera.it/parlam/leggi/deleghe/01031dl.htm.

[24] Gaglioti S., Infusino E., Caloiero T., Callegari G., Guagliardi I. (2019). Geochemical Characterization of Spring Waters in the Crati River Basin, Calabria (Southern Italy). Geofluids.

[25] Guagliardi I., Caloiero T., Infusino E., Callegari G., Ricca N. (2021). Environmental Estimation of Radiation Equivalent Dose Rates in Soils and Waters of Northern Calabria (Italy). Geofluids.

[26] Balestrieri A., Remonti L., Smiroldo G., Prigioni C., Reggiani G. (2008). Surveying otter Lutra Lutra distribution at the southern limit of its Italian range. Hystrix It. J. Mamm..

[27] Iovine G., Guagliardi I., Bruno C., Greco R., Tallarico A., Falcone G., Lucà F., Buttafuoco G. (2017). Soil-gas radon anomalies in three study areas of Central-Northern Calabria (Southern Italy). Nat. Hazards.

[28] Bonardi G., Cavazza W., Perrone V., Rossi S., Vai G.B., Martini I.P. (2001). Calabria-Peloritani terrane and northern Ionian Sea. Anatomy of an Orogen: The Apennines and Adjacent Mediterranean Basins.

[29] Vignaroli G., Minelli L., Rossetti F., Balestrieri M.L., Faccenna C. (2012). Miocene thrusting in the eastern Sila Massif: Implication for the evolution of the Calabria-Peloritani orogenic wedge (southern Italy). Tectonophysics.

[30] Le Pera E., Arribas J., Critelli S., Tortosa A. (2001). The effects of source rocks and chemical weathering on the petrogenesis of siliciclastic sand from the Neto River (Calabria, Italy): Implications for provenance studies. Sedimentology.

[31] Liotta D., Caggianelli A., Kruhl J.H., Festa V., Prosser G., Langone A. (2008). Multiple injections of magmas along a Hercynian mid-crustal shear zone (Sila Massif, Calabria, Italy). J. Struct. Geol..

[32] Critelli S., Muto F., Tripodi V., Perri F., Schattner U. (2011). Relationships between lithospheric flexure, thrust tectonics and stratigraphic sequences in foreland setting: The Southern Apennines foreland basin system, Italy. New Frontiers in Tectonic Research at the Midst of Plate Convergence.

[33] Scarciglia F., Critelli S., Borrelli L., Coniglio S., Muto F., Perri F. (2016). Weathering profiles in granitoid rocks of the Sila Massif uplands, Calabria, southern Italy: New insights into their formation processes and rates. Sediment. Geol..

[34] Guagliardi I., Cicchella D., Rosa R. (2012). A Geostatistical approach to assess concentration and spatial distribution of heavy metals in urban soils. Water Air Soil Pollut..

[35] Fabbricatore D., Robustelli G., Muto F. (2014). Facies analysis and depositional architecture of shelf-type deltas in the Crati Basin (Calabrian Arc, south Italy). Ital. J. Geosci..

[36] Gallo M., Iovino F., Agnoletti M., Anderson S. (2000). A brief history of the forest changes in the Sila Greca mountains. Forest History International Studies on Socioeconomic and Forest Ecosystem Change. Report No. 2 of the IUFRO Task Force on Environmental Change.

[37] Beck H.E., Zimmermann N.E., McVicar T.R., Vergopolan N., Berg A., Wood E.F. (2018). Present and future Köppen-Geiger climate classification maps at 1-km resolution. Sci. Data.

[38] Coscarelli R., Gaudio R., Caloiero T., Rodda J.C., Ubertini L. Climatic trends: An investigation for a Calabrian basin (southern Italy). Proceedings of the International Symposium The Basis of Civilization.

[39] Buttafuoco G., Caloiero T., Ricca N., Guagliardi I. (2018). Assessment of drought and its uncertainty in a southern Italy area (Calabria region). Measurement.

[40] Bianucci G., Ribaldone E., Bianucci E., Ulrico H. (1985). Composizione Chimica Delle Rocce, La Chimica Delle Acque Sotterranee.

[41] Appelo C.A.J., Postma D. (2005). Geochemistry. Groundwater and Pollution.

[42] Ako A.A., Shimada J., Hosono T., Kagabu M., Ayuk A.R., Nkeng G.E., Takem G.E.E., Takounjou A.L.F. (2012). Spring water quality and usability in the Mount Cameroon area revealed by hydrogeochemistry. Environ. Geochem. Health.

[43] Callegari G., Cantasano N., Infusino E., Callegari G., Cipriani M.G. (2012). Hydrogeological and geochemical assessment of some spring waters in the municipal area of Chiaravalle Centrale (Calabria, southern Italy). Rend. Online Soc. Geol. Ital..

[44] AWWA (1975). AWWA standard for asbestos-cement transmission pipe, 18 in. through 42 in., for water and other liquids. J. Am. Water Works Assoc..

[45] AWWA (1980). AWWA Standard for Asbestos-Cement Distribution Pipe. 4 in. Through 16 in. (100 mm Through 16 in.) (100 mm Through 400 mm). https://engage.awwa.org/PersonifyEbusiness/Store/Product-Details/productId/18631.

[46] Collins W.D. (1923). Graphic representation of water analyses. Ind. Eng. Chem..

[47] Piper A.M. (1944). A graphic procedure in the geochemical interpretation of water-analyses. Trans. AGU.

[48] Langelier W.F., Ludwig F. (1942). Graphical methods for indicating the mineral character of natural waters. J. Am. Water Work. Assoc..

[49] Cao W.G., Yang H.F., Liu C.L., Li Y.J., Bai H. (2018). Hydrogeochemical characteristics and evolution of the aquifer systems of Gonghe Basin, Northern China. Geosci. Front..

[50] Ravikumar P., Prakash K.L., Somashekar R.K. (2013). Evaluation of water quality using geochemical modeling in the Bellary Nala Command area, Belgaum district, Karnataka State, India. Carbonates Evaporites.

[51] Rao N.S., Subrahmanyam A.S., Kumar S.R., Srinivasulu N., Rao G.B., Rao P.S., Reddy G.V. (2012). Geochemistry and quality of groundwater of Gummanampadu sub-basin, Guntur District, Andhra Pradesh, India. Environ. Earth Sci..

[52] Rao N.S., Vidyasagar G., Rao P.S., Bhanumurthy P. (2017). Chemistry and quality of groundwater in a coastal region of Andhra Pradesh, India. Appl. Water Sci..

[53] Waters N.M., Coffey W.J. (1988). Expert Systems and Systems of Experts, Chapter 12. Geographical Systems and Systems of Geography.

[54] Hutchinson M.F., Gessler P.E. (1994). Splines—More than just a smooth interpolator. Geoderma.

[55] Apaydin H., Sonmez F.K., Yildirim Y.E. (2004). Spatial interpolation techniques for climate data in the GAP region in Turkey. Clim. Res..

[56] Mueller D.K. (1995). Nutrients in Ground Water and Surface Water of the United States: An Analysis of Data through 1992.

[57] Infusino E., Callegari G., Cantasano N. (2016). Release of nutrients into a forested catchment of southern Italy. Rend. Fis. Acc. Lincei..

[58] Padilla F.M., Gallardo M., Manzano-Agugliaro F. (2018). Global trends in nitrate leaching research in the 1960–2017 period. Sci. Total Environ..

[59] Accardo V., Angeletti V., Cocozziello B., D’Arienzo V., Aquino De Gennaro V., De Angelis C., De Rosa E., Di Meo T., Imperatrice M.I., Mainolfi P. Il monitoraggio dell’inquinamento idrico da nitrati degli acquiferi della Campania. Proceedings of the Conference Accettabilità Delle Acque per usi Civili e Agricoli.

[60] Wilcox J.C., Holland W.D., McDougald J.M. (1956). Relation of elevation of a mountain stream to reaction and salt content of water and soil. Can. J. Soil Sci..

[61] Callegari G., Frega G., Infusino E. Deposizioni atmosferiche nell’area urbana di Cosenza in Calabria. Proceedings of the Conference Qualità Dell’aria Nelle Città Italiane.

[62] Guo B., Yang F., Wu H., Zhang R., Zang W., Wei C., Zhang H. (2021). How the variations of terrain factors affect the optimal interpolation methods for multiple types of climatic elements?. Earth Sci. Inform..

[63] Kazemi E., Karyab H., Mohammad-Mehdi Emamjome M. (2017). Optimization of interpolation method for nitrate pollution in groundwater and assessing vulnerability with IPNOA and IPNOC method in Qazvin plain. J. Environ. Health Sci. Engineer..

[64] Zabihi M., Pourghasemi H.R., Pourtaghi Z.S., Behzadfar M. (2016). GIS-based multivariate adaptive regression spline and random forest models for groundwater potential mapping in Iran. Environ. Earth Sci..

[65] Naghibi S.A., Moradi D.M. (2017). Evaluation of four supervised learning methods for groundwater spring potential mapping in Khalkhal region (Iran) using GIS-based features. Hydrogeol. J..

